# Artificial Intelligence Powers Protein Functional Annotation

**DOI:** 10.1002/advs.202524373

**Published:** 2026-04-07

**Authors:** Wenkang Wang, Qiurong Yang, Min Zeng, Ruiqing Zheng, Min Li

**Affiliations:** ^1^ School of Computer Science and Engineering Central South University Changsha China

**Keywords:** artificial intelligence, enzyme commission, functional annotation, gene ontology, protein function

## Abstract

Protein functional annotation is essential for understanding biological processes, disease mechanisms, and enzyme activities, yet experimental validation remains costly and low‐throughput. With the rapid development of Artificial Intelligence (AI), a wide range of computational approaches have been proposed to infer protein functions. This review systematically examines methods for annotating Gene Ontology (GO) terms and Enzyme Commission (EC) numbers. These are two complementary systems that capture different aspects of protein functions. Based on these two systems, we first synthesize existing approaches into six general modeling paradigms with a clear, structured framework. Then, we introduce GO and EC in a parallel manner, consisting of representative methods, commonly used evaluation metrics, prediction scenarios, and task‐specific challenges. Finally, we outline emerging opportunities and future directions aimed at achieving more accurate, context‐dependent, and high‐resolution protein functional annotation.

## Graphical abstract

This review systematically summarizes how artificial intelligence advances protein functional annotation. It organizes existing methods into six unified modeling paradigms and analyzes their applications in Gene Ontology and Enzyme Commission prediction. The authors highlight current challenges, emerging multimodal and language model approaches, and future directions toward accurate, context‐aware, and automated protein function annotation systems.

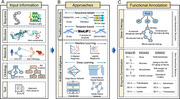

## 1 Introduction

Proteins orchestrate fundamental life processes, ranging from transcriptional regulation and catalysis to molecular transport. Functional annotation of proteins is essential for elucidating pathogenic mechanisms and accelerating drug discovery [1]. However, while genomic sequencing and structure prediction technologies have exponentially expanded the number of known protein sequences and structures, fewer than 0.1% of proteins possess experimentally validated functional annotations [2]. Given that experimental characterization is expensive and low‐throughput, large‐scale protein function annotation heavily relies on computational approaches [3].

However, computational inference of protein function remains challenging [4]. First, the label space of protein functions is vast, where each protein is associated with only a few functions, resulting in severe sparsity and a pronounced long‐tail distribution dominated by rare labels. Furthermore, sequence identity is often insufficient for functional similarity, particularly in case of remote homology, where traditional sequence alignment‐based and template‐based methods struggle to generalize [5].

In recent years, advanced Artificial Intelligence (AI) technologies, especially machine learning and deep learning, have demonstrated great potential for biological knowledge representation [6, 7]. More and more AI‐based computational approaches have been proposed for protein functional annotation [8, 9, 10, 11].

Currently, Gene ontology (GO) and Enzyme Commission (EC) are the most established systems for functional annotation [12, 13, 14]. The GO knowledge encompasses all known functions of proteins, spanning from the molecular to the organism level across diverse species. These functions are represented as GO terms and categorized into three sub‐ontologies: Biological Process (BP), Molecular Function (MF), and Cellular Component (CC). In each sub‐ontology, GO terms are organized as nodes within a Directed Acyclic Graph (DAG), where edges denote specific functional relationships (e.g., “is‐a”, “part‐of”). This architecture forms a loose hierarchical structure, where child nodes are more specialized than their parent nodes. In contrast, the EC system focuses exclusively on enzymes. It utilizes four digits to specify catalytic functions, forming a strict hierarchy. As more digits are known in EC number, the corresponding catalytic function becomes increasingly specific. In summary, while EC numbers denote specific catalyzed reactions of enzymes, GO terms describe broader biological contexts. These complementary perspectives offer valuable insights into the diverse protein functions.

The rapid development of AI‐based function prediction has catalyzed diverse cutting‐edge models that span a wide range of biological knowledge, from sequence and structure to protein interactions, pathways, and biomedical literature [15, 16, 17]. However, these advances have emerged across different data modalities and methodological paradigms, resulting in a highly fragmented landscape that is difficult to navigate or systematically compare [4]. Furthermore, existing surveys typically focus on either GO term or EC number prediction in isolation, rarely analyzing these two fundamental annotation tasks together [18, 19, 20]. This absence of an integrated, paradigm‐centric perspective limits our understanding of how AI‐based models operate across annotation systems and how different modeling strategies interrelate. Therefore, a comprehensive survey that jointly organizes GO and EC prediction within a unified methodological framework is essential.

**FIGURE 1:**
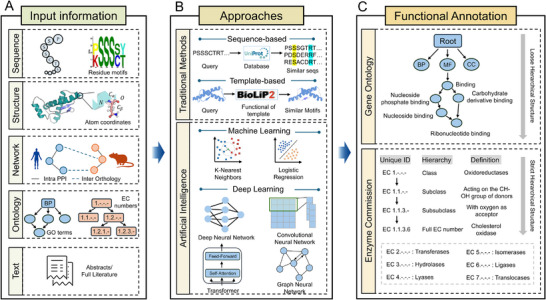
The computational pipelines of protein functional annotation. This figure illustrates the three key steps in protein functional annotation, including: (A) Modal information of available proteins used for function prediction, consisting of protein sequence, protein structure, protein networks, biological ontology, and literature text. (B) Traditional and AI‐based Approaches. (C) Two functional annotation systems (GO terms and EC numbers).

This review offers an integrated, structured synthesis of AI methodologies for protein functional annotation, bridging the GO and EC systems (Figure 1). We first synthesize existing approaches into six major modeling paradigms with a clear, structured framework: sequence‐based, structure‐based, network‐based, ontology‐based, multimodal integration‐based, and Large Language Model (LLM)‐based. These paradigms illustrate how different data modalities and representation strategies contribute to functional annotation. Building on these frameworks, we systematically review GO term predictors and EC number predictors in parallel, covering methodological developments, practical prediction cases, corresponding persistent challenges, commonly used annotation resources, and evaluation metrics. Finally, we outline emerging opportunities for unified, ontology‐aware functional modeling. We propose that integrating GO, EC, and broader pathway knowledge into a shared semantic space, potentially enabled by multimodal learning and next‐generation protein foundation models. Concurrently, with the growth of AI, developing LLM‐based agents are expected to open new avenues for large‐scale and accurate protein function annotation.

## 2 Computational Frameworks for AI‐Based Protein Functional Annotation

**FIGURE 2:**
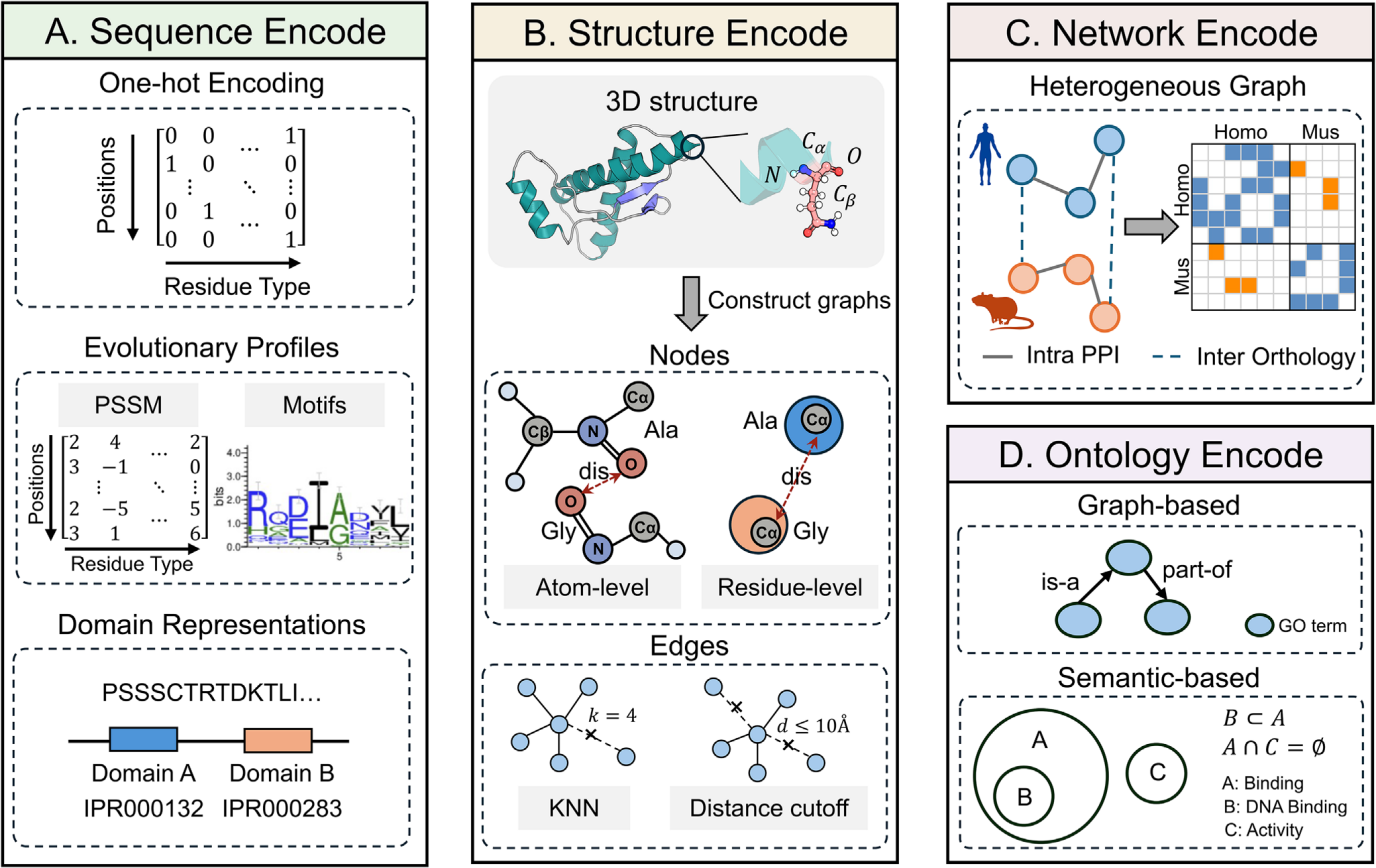
Data encoding schemes for different modalities, including: (A) protein sequence encoding methods. (B) protein structural graph construction. (C) heterogeneous protein network construction. (D) biological ontology modeling methods.

**FIGURE 3:**
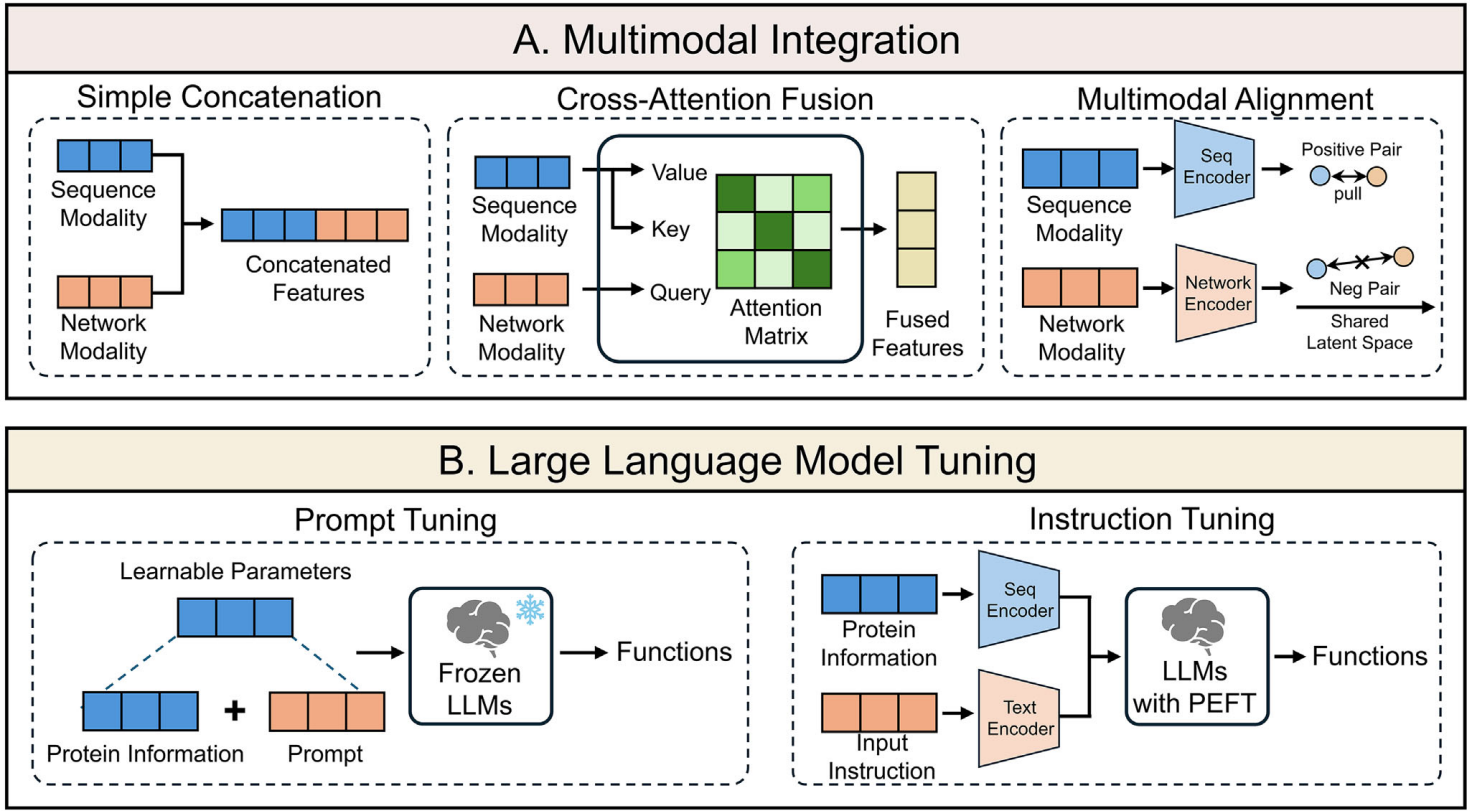
Strategies frequently used in multimodal integration and LLM tuning. (A) The details of three commonly used architectures for multimodal integration. (B) The details of tuning existing LLMs.

Benefiting from advancements in high‐throughput sequencing technology and AI‐driven protein structure research, vast amounts of biological data have been explored, driving the emergence of diverse computational approaches. Current AI‐based methods construct various frameworks for protein functional annotation, distinguished by their underlying assumptions, input modalities, and model architectures. To provide conceptual clarity, we categorize these approaches into several computational frameworks (Figures 2 and 3). Each framework adopts a unique perspective to decipher the associations between proteins and their functions, reflecting a specific mode of biological reasoning (Table 1).

**TABLE 1:** Strengths and Limitations Across Paradigms.

Paradigm	Data Requirements	Computation Cost	Interpretability	Specific Scenarios
Sequence‐based	Protein sequence	Moderate (CNN and Transformer)	Significance analysis at motif‐ or residue‐level	Suitable for large‐scale benchmarking, as they only require sequences.
Structure‐based	Protein sequences and structures	High (GNN‐based models and geometric learning)	Detection of key residues, spatial interactions, and pocket structures.	Effective for remote homologs and EC‐related tasks. Limited for IDPs or low‐confidence structures.
Network‐based	PPI or homology or functional association networks	Moderate (Network topology learning) High (GNN‐based models)	Detection of functional modules or key associations	Effective for inferring intra‐species functions. Limited for orphan proteins or poorly characterized species.
Ontology‐based	Label hierarchies (GO/EC graph)	Moderate (GNN‐based models and semantic embedding)	Exploring the relationship between functions	Effective for unseen or low‐frequency labels. Complementary to other paradigms.
Multimodal‐based	Protein sequences, structures, networks and ontologies	High	Variable (depends on modality fusion strategies)	Effective when multiple data sources are available. Limited by sparse‐data input.
LLM‐based	Multimodal LLMs and corresponding textual prompts	Very high (Retraining) High (Fine tuning)	Natural language explanations but lack mechanistic grounding	Flexible across tasks. Risk of hallucination.

### 2.1 Sequence‐Based Framework

Protein sequences, as the most abundant and accessible biological source, are intrinsically linked to protein structures and functions [21]. Currently, annotating protein functions from protein sequences remains the most widely used framework. These methods extract high‐dimensional feature embeddings directly from amino acid sequences to infer the corresponding functions.

To adapt protein sequences for computational modeling, diverse encoding strategies have been developed, including one‐hot encoding, evolutionary profiles [22, 23, 24], and domain‐based representation [25, 26] (Figure 2A). Specifically, one‐hot encoding treats each amino acid as a discrete token, analogous to vocabulary in Natural Language Processing (NLP). Evolutionary profiles characterize sequences from a large biological history. It aligns the target protein sequence against extensive databases, capturing conservation patterns, and substitution probabilities at each position. Domain‐based representation incorporates family [27], domains [28], and functional sites [29] information. It maps protein sequence fragments to specific families, predicts domains and important sites, and encodes all this information as unique identifier (e.g., InterPro entry [26]).

Building upon these sequence encoding schemes, diverse deep learning architectures are adopted to extract high‐level semantic features from sequences. For example, Deep Neural Networks (DNNs) [30] often serve as embedding layers to project sparse one‐hot representations of amino acids and domains into dense vectors, facilitating the capture of deep non‐linear relationships. Additionally, Convolutional Neural Networks (CNNs) [31] are widely used to extract local patterns, while Recurrent Neural Networks (RNNs) [32] and Long Short‐Term Memory networks (LSTMs) [33] exhibit remarkable abilities in modeling sequential and long‐range patterns. Notably, recent transformer‐based protein Large Language Models (pLLMs), such as ESM [34] and ProtT5 [35], have revolutionized this field. These models demonstrate superior efficacy in learning contextualized protein representations, achieving state‐of‐the‐art performance across diverse downstream tasks, including functional annotation.

Overall, the ubiquity of large‐scale sequence data renders sequence‐based frameworks broadly applicable. However, these methods struggle when sequence similarity is low (e.g., orphan proteins). These limitations underscore the critical need to incorporate higher‐order biological information beyond sequences.

### 2.2 Structure‐Based Framework

As the higher‐order realization of sequences, protein structures provide explicit geometric and physicochemical constraints, serving as direct determinants of protein function. Structure‐based approaches primarily leverage spatial information to construct residue‐level or atom‐level interaction networks, capturing contact residue patterns and the geometry surrounding key sites to annotate protein functions.

Current structure encoding strategies primarily involve constructing graphs at different levels, including atom‐level and residue‐level (Figure 2B). In residue‐level models, each amino acid is treated as a single node based on their $C_{\alpha }$ or $C_{\beta }$ atoms, whereas atom‐level models treat all atoms as distinct nodes to capture fine‐grained interactions. Furthermore, PDB graphs are constructed using the K‐Nearest Neighbors (KNN) algorithm or a fixed distance threshold. Crucially, to ensure the E(3) equivariant of protein structures, coordinate information is often utilized to construct relative position representations for different atoms within amino acids as node features and relative position representations for different nodes as edge features.

Building upon these PDB graphs, Graph Neural Networks (GNNs) [36, 37] are the most widely used architectures for capturing structural geometry, such as graph convolutional networks (GCNs) [38], graph attention networks (GATs) [39], and geometric vector perceptrons (GVPs) [40]. Following the iterative propagation and aggregation of neighbor features, pooling operations are employed to integrate amino acid‐level features into protein‐level features for functional annotations. These pooling strategies are generally categorized into standard global pooling (e.g., mean, max, and adaptive pooling) and biological knowledge‐guided pooling. While standard approaches are computationally efficient, they often lack interpretability [41]. Conversely, biological knowledge‐guided pooling leverages biological features to selectively merge critical functional regions (e.g., active sites), thereby enhancing both predictive accuracy and model explainability [10].

Unlike sequence‐based methods, structure‐based approaches can directly reveal key functional regions, such as catalytic or binding sites. However, their performance is constrained by the quantity of structural data. Furthermore, failing to consider interactions and contextual information also limits the performance of these methods. It would be more robust to incorporate more multimodal information.

### 2.3 Network‐Based Framework

Many protein functions arise not from individual molecules but from their participation in larger interaction networks [42, 43]. Proteins that interact physically or functionally often participate in shared pathways or biological processes, thus making network topology highly informative for functional inference, which is different from methods relying on sequence or structural similarity. Network‐based frameworks treat the PPI graph itself as the inference engine: proteins are represented as nodes, interactions are denoted as edges, and functional features propagate through local neighborhoods. Specifically, these approaches typically encode individual protein attributes (e.g., sequence and structure) as node features, construct multi‐type edges based on different types of interaction relationships (e.g., co‐evolution, physical interaction, co‐expression, and regular) [44], aggregate information from neighboring proteins through network topology, and infer corresponding functions. Notably, protein interactions only exist within the same species, which means that different species form independent PPI networks, and information cannot be transferred between species. To bridge these disconnects, orthology relations and network alignment technologies are applied to connect proteins across different species [45] (Figure 2C).

To extract functional insights from these heterogeneous topologies, various GNN architectures are employed. Beyond the previously mentioned GCNs and GATs, Heterogeneous Graph Neural Networks (HGNNs) are also utilized here, as they can effectively distinguish and integrate diverse interactions between proteins [46].

In summary, network‐based modeling excels in scenarios where functional identity is driven by interaction context rather than sequence or structure alone, offering particular utility for annotating remote homology. However, its performance remains constrained by the coverage, quality, and noise of interactions from high‐throughput technologies. Additionally, solely relying on interactions cannot resolve functions at residue‐level. Consequently, network‐based approaches will be more powerful when integrated with sequence, structural, or ontology‐aware paradigms within a unified framework.

### 2.4 Ontology‐Based Framework

As mentioned before, functional annotations in GO and EC are not discrete labels but rather well‐defined hierarchical ontologies that encode semantic relationships among functions. These hierarchical structures consist of rich biological dependencies, such as parent–child inclusion and common ancestors, which are crucial for inferring unknown functions based on known ones [47]. Ontology‐aware models explicitly incorporate these semantic constraints into the AI framework, ensuring that predictions are consistent with the structure of ontologies. This framework enables more reliable inference of both broad and specific functional annotations.

Current strategies for ontology modeling generally follow two paradigms (Figure 2D): graph‐based modeling with relational constraints and semantic‐based geometric embedding [48]. In graph‐based approaches, GO terms and EC numbers are treated as nodes in a directed acyclic graph (DAG), with edges representing their relationships. Alternatively, semantic‐based approaches represent each function as a geometric object in a high‐dimensional space, encoding each relationship type to ensure that the spatial arrangement of these geometric objects rigorously satisfies the relational constraints (e.g., logical entailment or set inclusion) defined by the ontology.

Based on these modeling approaches, existing AI architectures employ distinct mechanisms to refine functional representations. In graph‐based models, GNNs are commonly employed to update the representations of functions across the DAG, preserving the hierarchical structures of ontologies [49]. In contrast, semantic‐based approaches often leverage simple embedding layers with DNNs to project functions and their relationships into continuous embedding spaces, ensuring that similar functions are placed close to each other [50]. A crucial aspect of these models is the incorporation of semantic loss functions. It penalizes deviations from expected hierarchical relationships in GO and EC, ensuring consistency with the biological structure of the ontologies.

Ontology‐based frameworks play a critical role in rationalizing predictions, reducing logical inconsistencies to ensure biological plausibility. These methods excel in scenarios involving long‐tail function distributions and rare functions. However, their performance is restricted by the completeness and accuracy of the ontology background. Notably, ontology‐aware modules are highly composable. They can be easily integrated with other protein‐centric frameworks to further enhance the accuracy of functional annotation.

### 2.5 Multimodal Integration‐Based Framework

Protein function is determined not only by their sequences and structures but also by their interactions and corresponding environment. Relying on a single data modality (e.g., sequence‐based or network‐based) is insufficient for accurate functional annotation and is susceptible to data noise. Integrating multiple biological sources provides a more comprehensive understanding of protein function.

For instance, sequence‐based methods capture evolutionary and sequence motifs, structure‐based models provide detailed geometric features, and protein–protein interaction (PPI) networks encode functional context within cellular networks. Integrating these modalities allows for a richer, more robust functional representation, which is crucial for accurately predicting protein functions.

To comprehensively and accurately fuse multiple representations, beyond naive feature concatenation, several more efficient methods have been proposed (Figure 3A). For example, cross‐attention mechanisms [51] utilize information from one modality to guide the adaptive weighting and refinement of another, effectively modeling the inter‐dependencies between data streams. Concurrently, contrastive learning strategies are increasingly employed to align features across multiple modalities [52]. By maximizing the agreement between different views of the same protein, these methods integrate multimodal data into a shared, semantically coherent latent space.

Despite its promising potential, multimodal integration remains challenging due to the difficulty of aligning heterogeneous data types. There is still an urgent need to propose effective strategies to facilitate efficient interactions between different types of data. Furthermore, some biological modalities, such as transcriptomic profiles, remain underutilized, limiting the performance of multimodal models. Nonetheless, multimodal integration frameworks remain a significant direction for improving the accuracy and robustness of protein functional annotation.

### 2.6 Large Language Modle‐Based Framework

Recent breakthroughs in natural language processing (NLP) have catalyzed the emergence of powerful foundation language models (e.g., LLaMA [53], GPT [54], DeepSeek [55]). Trained on massive corpora of general text, these models have demonstrated outstanding performance across question‐answering tasks in diverse domains, showcasing robust capabilities in knowledge representation, summarization, and reasoning. Fine‐tuning existing large language models (LLMs) can transform them into specialized experts for understanding protein sequences, structures, and functions, offering novel approaches to protein functional annotation [56].

Current LLM‐based frameworks typically employ prompt engineering [57] or instruction tuning [58] strategies to adapt the model to protein‐specific contexts (Figure 3B). In this paradigm, the model is conditioned on protein descriptors (e.g., sequences or textual descriptions) via specific prompts to annotate functions [59]. For instance, a standard description might be “The protein sequence is QVGAAS…” with a task‐specific prompt “Describe the function of this protein” or “What type of enzyme is this? Select from the following options: hydrolase, oxidoreductase, transferase…”. This approach enables existing LLMs to output structural information, such as GO terms or enzyme types.

However, due to the large scale of model parameters, directly fine‐tuning model weights is often impractical. To address this, Parameter‐Efficient Fine‐Tuning (PEFT) strategies have emerged as the standard solution [60, 61]. These frameworks typically combine small‐scale or pre‐trained protein encoders to extract protein embeddings, then introduce only a small set of trainable parameters to perform light fine‐tuning on the frozen LLM backbone. This ensures the efficiency and effectiveness of model updates, allowing LLMs to focus on functional annotation tasks while retaining their general knowledge.

Despite their promising potential, LLM‐based frameworks still face numerous challenges. The performance of these models heavily relies on the quality of fine‐tuning data and the structure of the prompts used. Additionally, when protein information becomes complex, the number of token inputs increases significantly. This may prevent LLMs from being applied to long proteins, and the inference time will also be substantially higher than that of previously introduced frameworks. Nevertheless, LLM‐based frameworks represent a major paradigm shift in protein functional annotation, providing a new direction for integrating NLP techniques with molecular biology tasks.

## 3 Gene Ontology Prediction

In this section, we examine GO prediction methods through the modeling frameworks introduced in Section 2. The details of existing methods can be obtained from Table 2. We discuss how different methods leverage protein sequences, structures, or other biological knowledge to address GO annotation according to our predefined encoding schemes and feature representation architectures. Furthermore, we introduce prediction scenarios and current challenges, providing a comprehensive perspective on the current landscape of GO term prediction.

**TABLE 2:** The details of existing methods of GO term prediction.

Methods	Encode Schemes & Model Architecture							Key Advantages	Potential Limitations
	Sequence	Structure	Network	Ontology	Fusion	LLM	Others		
DeepGO [62]	3‐mer one‐hot: 1D‐CNN & MaxPooling	/	/	DAG: hierarchical classification	/	/	/	The earliest framework integrating DNN with PPI networks for GO term prediction. Combines sequence‐derived features with network embeddings to improve functional inference.	Strongly dependent on the quality of PPI networks. CNN‐based architecture limits modeling of long‐range sequence dependencies. Reduced performance for rare or sparsely annotated GO terms.
DeepGOPlus [63]	One‐hot: multi‐1D‐CNN & MaxPooling	/	/	/	/	/	/	Relies solely on sequences. Integrates CNNs with sequence similarity search (DIAMOND), improving predictive accuracy.	Still partially dependent on homologous sequence similarity. Limited performance for remote homology.
TALE [64]	One‐hot: Transformer Encoder	/	/	one‐hot: 1D‐CNN	Multiplication	/	/	Employs Transformer architecture to capture long‐range dependencies in protein sequences. Incorporates hierarchical label embeddings that reflect the GO ontology structure.	Limited to sequence‐derived one‐hot information.
ATGO [65]	One‐hot: Transformer‐based LLM Architecture	/	/	/	/	/	/	Built upon pLLMs (ESM‐1b), enabling rich contextual representations. Adaptive fusion strategies demonstrate improved generalization to remote homology.	High computational resource requirements.
AnnoPRO [69]	Evolutionary Profiles: DNN & Multichannel CNN	/	/	/	/	/	/	Transforms protein sequences into image‐like representations for multimodal deep learning. Addresses long‐tail GO term prediction.	Complex feature transformation pipeline. Potential loss of fine‐grained sequential information.
DeepFRI [72]	One‐hot: pre‐trained LSTM language model	Residue/Atom‐distance/KNN: GCN	/	/	Mean Pool	/	/	Employs GCNs on protein contact maps. Provides residue‐level interpretability.	Requires experimentally determined or accurately predicted 3D structures. Sensitive to structural noise and prediction errors.
GAT‐GO [73]	One‐hot: pre‐trained ESM‐1b & 1D‐CNN Evolutionary Profiles: 1D‐CNN	Residue‐distance: GAT	/	/	Self‐Attention Graph Pool	/	/	Utilizes GATs to dynamically weight neighboring residues.	Attention mechanisms increase computational cost.
GGN‐GO [74]	One‐hot: pre‐trained ESM2 & ProtT5	Residue‐distance: GVP	/	/	Self‐Attention Graph Pool	/	/	Proposes a geometric graph network capturing multi‐scale structural features (atomic + residue levels) and uses geometric vector representations for improved feature propagation.	Requires reliable structural/geometric features. Computational cost may increase due to multi‐scale geometric processing.
GPSFun [75]	One‐hot: pre‐trained ProtT5	Residue‐distance: Geometric GAT	/	/	Self‐Attention Graph Pool	/	/	A geometry‐aware server that combines language models (sequence embeddings) and geometric deep learning	Relies on predicted 3D conformations/geometric features
Struct2GO [76]	One‐hot: Node2Vec & pre‐trained SeqVec	Residue‐distance: GCN	/	/	Top‐k Rank Pooling Layer	/	/	Combines protein structure and sequence data to enhance prediction precision and generality.	Less applicable when structure is unavailable or low‐confidence
DPFunc [10]	One‐hot: pre‐trained ESM‐1b Domain: DNN	Residue‐distance: residual GCN	/	/	Domain‐guided Cross Attention	/	/	Use domain‐guided structure information improve prediction accuracy. Claims improved inference for unseen proteins with low sequence identity. Provides structure–function interpretability.	High data preparation costs which depends on structural/domain guidance inputs.
DeepGraphGO [17]	Domain: DNN	/	PPI: residual GCN	/	/	/	/	Integrates Interpro features with PPI graph representations. Applies graph convolution for functional information propagation.	Dependent on network completeness. Reduced accuracy for sparsely connected proteins.
DeepFMB [81]	One‐hot: pre‐trained ESM‐1b Domain: DNN	/	PPI: GCN Orthology: GCN	/	Linear Attention	/	/	Multimodal framework integrating sequence, PPI network and homology network. Employs multi‐branch neural architecture for feature fusion. Addresses limitations such as ignoring proteins lacking interactions.	Model complexity increases training difficulty. Sensitive to missing modalities.
DeepPFP‐CO [81]	One‐hot: multi‐1D‐CNN & Bi‐LSTM Domain: DNN	/	PPI: DeepWalk	DAG: GCN	Concatenation & GCN	/	/	•	•
NetQuilt [78]	One‐hot: Blast	/	PPI: IsoRank	/	/	/	/	Enables cross‐species knowledge transfer through network embeddings.	Cross‐species network alignment can be challenging. Dependent on interspecies network similarity.
DeepGOA [67]	Domain: DNN One‐hot: Word2Vec & LSTM	/	PPI: Deepwalk	/	Concatenation	/	/	Extracts sequence semantic/subsequence features and PPI topological features	Depends on PPI network coverage/quality
GNNGO3D [49]	One‐hot: pre‐trained ESM‐1b Evolutionary Profiles: 1D‐CNN	Residue‐distance: GAT	/	DAG: GCN	SAGPool & Mean Pool & Concatenation	/	/	Combines protein sequence + tertiary structure together with GO term hierarchy information for function prediction	Requires reliable tertiary structure information. Computational costs is typically higher for 3D structure and hierarchy modeling.
PU‐GO [82]	One‐hot: pre‐trained ESM2	/	/	DAG: PU loss constraint	Concatenation	/	/	Formulates function prediction as a positive–unlabeled ranking problem and uses ontology‐based priors.	PU risk minimization depends on assumptions about unlabeled annotations and class priors. Performance can be sensitive to prior estimation.
DPGOK [83]	One‐hot: pre‐trained ESM2	/	/	DAG: Knowledge graph	Protein‐specific sigmoid	/	/	Proposes fusing GO knowledge with protein features for improved function prediction.	The high computational cost of integrating GO structural information with protein‐specific representations.
DeepGOZero [50]	Domain: DNN	/	/	Semantic‐based: EL Embedding	/	/	/	Implements zero‐shot learning based on GO ontology axioms. Enables prediction of GO terms absent from training data.	Performance is constrained by how informative/complete the ontology axioms and learned embeddings are.
DeepGO‐SE [8]	One‐hot: pre‐trained ESM2	/	/	Semantic‐based: EL Embedding	/	/	/	Incorporates pLLMs (ESM2) with DeepGOZero.	Increased methodological complexity compared with standard multi‐label classifiers.
NetGO [16]	One‐hot: BlastKNN 3mer one‐hot: LR Domain: LR Evolutionary Profiles: ProFET & LR	/	PPI: KNN	/	Learning To Rank	/	Naïve GO	Integrates sequence and massive network information under a learning‐to‐rank framework for large‐scale AFP.	May be less effective when network data are missing for query proteins.
NetGO 2.0 [122]	One‐hot: BlastKNN & RNN 3mer one‐hot: LR Domain: LR	/	/	/	Learning To Rank	/	Two additional classifiers: Naïve GO PubMed Literature: TF‐IDF & D2V & LR	Extends NetGO by incorporating massive sequence, text, domain/family and network information.	Increased dependence on heterogeneous data sources. Data availability become practical constraints.
NetGO 3.0 [123]	One‐hot: BlastKNN & pre‐trained ESM‐1b LR 3mer one‐hot: LR Domain: LR	/	/	/	Learning To Rank	/	Two additional classifiers: Naïve GO PubMed Literature: TF‐IDF & D2V & LR	Replaces a sequence component with pLLM embeddings (ESM‐1b).	Increased dependence on heterogeneous data sources. Data availability become practical constraints.
DeepMFFGO [84]	One‐hot: pre‐trained ESM‐1b & Transformer Evolutionary Profiles: DNN	Residue‐distance: GAT	/	DAG: Anc2vec	Transformer	Self‐learning Weighted Fusion	/	Proposes large‐scale multi‐feature fusion and aims to address bottlenecks in multi‐source fusion and GO hierarchy utilization.	Multi‐feature fusion frameworks can be sensitive to missing/noisy modalities.
ProtNLM [85]	Text description: simple tokenizer	/	/	/	/	T5 Language model	Unsupervised training & task‐specific fine‐tuning	Uses a T5‐style text generation framework to map protein sequences to natural language functional descriptions.	Outputs text descriptions: evaluation and calibration can be harder than fixed‐label classification. Generation models can introduce ambiguous phrasing requiring downstream normalization.
FAPM [87]	One‐hot: pre‐trained ESM2	/	/	Functional Text (prompt)	Q‐Former	Mistral‐7B	Three Training Objects: Protein ‐ Text Contrastive Protein ‐ Text Matching Protein ‐ Text Generation	A multimodal model linking natural language with protein representations for functional annotation.	Depends on the quality of multimodal alignment and prompt information. Require careful prompting for stable outputs.
ProteinChat [59]	Text description: fine‐tuning xTrimoPGLM‐1B	/	/	Prompt	Linear Adaptor	Vicuna‐13B	/	Multimodal LLM that takes protein sequence and generates function descriptions, including function labels and other related properties.	Requires task‐specific evaluation protocols. Reproducibility depends on training data and decoding settings.

### 3.1 Existing Methods

#### 3.1.1 Sequence‐Based Methods

Early approaches such as DeepGO [62] encode protein sequences using 3‐mer fragments and employ CNNs to capture local information relevant to function. DeepGOPlus [63] extends this idea by applying multi‐scale CNNs to one‐hot encoded sequences. Subsequently, leveraging the advantages of transformers in long text representation, models such as TALE [64] stack multilayer transformer architectures to derive contextualized representations from raw one‐hot inputs. Similarly, ATGO [65] leverages the pretrained protein language model ESM‐1b [66] as an encoder and applies contrastive learning to ensure that sequences with similar functions are represented similarly.

Additionally, evolutionary profiles such as PSSM and sequence domains also provide strong functional signals. DeepGOA [67] uses PSI‐BLAST [68] to generate evolutionary profiles PSSM, utilizes InterProScan [28] to detect sequence domians, and further leverages CNNs and DNNs to extract features for GO term prediction. AnnoPRO [69] also calculates various physicochemical features of sequences using PROFEAT [70, 71], such as amphiphilic pseudo amino acid composition, molecular interaction, quasi‐sequence‐order, and pseudo amino acid composition. By leveraging various feature similarity maps, AnnoPRO demonstrates that these database‐derived features enhance the accuracy of GO term prediction.

#### 3.1.2 Structure‐Based Methods

DeepFRI [72] is one of the earliest models to leverage PDB structures for GO term annotation. It constructs residue‐level contact graphs by defining nodes via $C_{\alpha }$ atoms and edges with 10 Å as distance threshold. After integrating residue features from pre‐trained language models and utilizing GCN to fuse sequential and structural information, DeepFRI employs mean pooling operations to represent protein features and annotate functions.

Furthermore, several methods, such as GAT‐GO [73], GGN‐GO [74], and GPSFun [75], utilize more effective GNN architectures to extract structural geometric features. For instance, GAT‐GO and GPSFun adopt graph attention mechanisms to integrate neighboring features with different weights. GGN‐GO uses GVPs to consider both $C_{\alpha }$ and $C_{\beta }$ atoms within residues. Moreover, GPSFun and GGN‐GO both integrate trainable parameters to pool residue features with learnable weights, which is more effective than previous mean pooling operations. Similarly, Struct2GO [76] adopts a self‐attention hierarchical pooling layer to select top‐k ratio residues [77]. Overall, these adaptive pooling operations assign different weights to amino acids, but they lack interpretability, and the learning process is difficult to optimize. Consequently, DPFunc [10] proposes a novel pooling strategy that leverages sequence domain information to learn amino acid weights. This approach not only effectively extracts protein‐level features but, more importantly, successfully identifies functionally active sites.

#### 3.1.3 Network‐Based Methods

DeepGraphGO [17] is a representative GNN‐based approach for GO annotation. It constructs PPI networks consisting of multiple species from the STRING database. With InterPro items as protein features, DeepGraphGO utilizes a residual GCN architecture to integrate interaction and sequence information, further annotating nodes (proteins).

However, one limitation is that PPI networks across different species are independent of each other, lacking connections between species. NetQuilt [78] uses IsoRank [79] to compute alignment scores between proteins from different species, forming a distance matrix with both intra‐species and inter‐species information. In Contrast, based on orthology relationships across different species [45], DeepFMB [80, 81] constructs two networks to represent inter‐species and intra‐species connections, respectively. It further integrates sequence representations from pLLMs to complete classification.

#### 3.1.4 Ontology‐Based Methods

At first, DeepGO [62] develops a hierarchical classification framework to predict GO terms, ensuring that the results satisfy the GO taxonomic structure for is‐a' relations. However, it requires huge memory resources, making it hard to apply to large‐scale GO terms. Recently, more and more methods utilize GNNs to extract the hierarchical features of GO terms. For example, GNNGO3D [49] first feeds the one‐hot encoding and adjacency matrix of GO terms into GCNs, then fuses them with protein features to predict the probability of each GO term. PU‐GO [82] proposes a ranking‐based loss relying on the GO hierarchical structure to reduce the error ratio on false unlabelled GO functions. Unlike previous approaches, DPGOK [83] establishes a new strategy enabling GO embeddings to be tailored to individual proteins, thereby reflecting protein‐specific functional relevance.

Another representative approach is DeepGOZero [50] and its upgraded version, DeepGO‐SE [8]. They learn GO embeddings using a semantic‐based approach (EL Embeddings [48]), ensuring that the distances between GO terms in high‐dimensional space satisfy their relational constraints. This strategy effectively enhances inference accuracy for rare and unknown GO terms.

#### 3.1.5 Multimodal Integration‐Based Methods

DeepGOA [67], as an early approach utilizing multiple types of biological data, integrates protein sequential information (domains and PSSM) with PPI networks for GO term prediction. More recently, GNNGO3D [49] incorporates protein sequence, structure, and gene ontology for functional annotation. However, these methods fuse multi‐type biological information through simple concatenation operations, limiting their performance. To address these challenges, NetGO [16] first trains a simple classifier for each modality, then uses the learning‐to‐rank framework to fuse the results from each modality and generate the final prediction. DPFunc [10] utilizes a modified cross attention mechanism to integrate domain and structural features. DeepMFFGO [84] proposes a self‐learning fusion strategy that performs weighted summation of sequential, structural, and ontology features.

#### 3.1.6 LLM‐Based Methods

ProtNLM [85] is a tool developed by Google for automatically annotating unannotated proteins in UniProtKB [86]. It leverages transfer learning to train a sequence‐to‐sequence language model based on the T5 architecture. It predicts or selects functions within a specified range based on input sequence information.

Recently, more methods have begun incorporating encoders for protein information, significantly enhancing the performance of existing models. For example, FAPM [87] encodes protein sequences using a pre‐trained pLLM (ESM2 [34]) and aligns protein embeddings with their corresponding functional text by fine‐tuning a pre‐trained Q‐Former model [88]. Finally, based on the output of Q‐Former, another pre‐trained LLM, Mistral‐7B [89], is utlized to generate protein functional descriptions. Furthermore, a stronger multimodal LLM, ProteinChat [59], has been proposed for functional annotation. It consists of a trainable protein sequence encoder and a trainable prompt text encoder, utilizing LoRA technology [90] to fine‐tune the LLM Vicuna‐13B [91], finally generating corresponding functional descriptions.

### 3.2 Prediction Scenarios

Beyond model design and evaluation metrics, the construction and partitioning of datasets significantly influence our assessment and selection of GO prediction methods, reflecting diverse scenario requirements. Here, we summarize commonly used dataset partitioning schemes, including time‐based splits, similarity‐controlled splits, and cross‐species splits.

Time‐based splits aim to simulate real‐world annotation trajectories by training on GO annotation data up to a preset deadline and evaluating on newly added annotations after that. This strategy is adopted in the Critical Assessment of Function Annotation (CAFA) [2], avoiding knowledge leakage while reflecting a model's ability to annotate unseen proteins.

Similarity‐controlled splits are designed to control homology by clustering proteins based on sequence identities and assigning clusters to either training or test sets. This strategy prevents closely related proteins from appearing in training and test data simultaneously [92]. Thus, it is able to evaluate models' generalization ability, assess their performance on proteins with low sequence similarity, and confirm whether models infer function based on protein information rather than overfitting specific sequence patterns.

Cross‐species splits aim to simulate newly sequenced or understudied species. In this scenario, models are trained on proteins from partially known species and tested on proteins from unseen species. This setup explores the models' knowledge transfer capabilities, which are crucial for functional annotation in bacteria with only sequence data available.

Notably, although temporal splitting is more reliable, it does not eliminate homologous relationships across different time periods and may result in distribution shifts due to sampling biases of newly characterized protein families. Therefore, we believe it is essential to combine both strategies. For instance, performing temporal splitting followed by sequence or structure similarity‐controlled splitting would enable a more rigorous and realistic evaluation of model performance.

### 3.3 Challenges

Despite substantial progress, GO term prediction remains a fundamentally challenging task due to several inherent data and methodological limitations.

A primary challenge is the incompleteness of functional annotations, which results in label noise. Since most proteins possess only a fraction of experimentally validated functions, treating unannotated terms as definitive negatives introduces massive false‐negative labels, which severely confuses the decision boundaries during model training. This is exacerbated by the long‐tail distribution of GO terms. While general terms near the root have abundant annotations, highly specific terms occur rarely. This imbalance typically leads to models that perform well on generic functions but fail to recall specific tail classes.

Additionally, from a modeling perspective, ensuring output consistency remains difficult. Standard deep learning models often fail to inherently respect the hierarchical dependencies of the GO DAG. Consequently, raw output logits frequently cannot align with the hierarchical dependencies among GO terms and and typically need additional post‐processing. Furthermore, cross‐species generalization poses a severe barrier. Models trained on well‐annotated species often struggle to transfer knowledge to remote species with sparse or biased annotations.

More importantly, predicting functions for Intrinsically Disordered Proteins (IDPs) or Intrinsically Disordered Regions (IDRs) represents a significant challenging in current methodologies. Unlike folded proteins, IDPs lack stable 3 D structures, rendering existing structure‐based encoders ineffective. Currently, there is a lack of specialized representation learning methods tailored for the conformations of IDPs, resulting in suboptimal performance when predicting functions related to signaling and regulation where disordered regions prevail.

**TABLE 3:** Systematic comparison of GO and EC annotations.

Aspect	Detail	GO term	EC number
Data	Label Structure	Directed acyclic graph (DAG)	Strict four‐level tree (EC x.x.x.x)
	Label Count	Very large (more than 10,000 terms)	Moderate (few thousand level‐4 EC numbers)
	Label Assignment	Multi‐label. Long‐tail distribution	Usually single catalytic pathway
	Preferred input data	Sequence. Structures. Networks. Ontology	Sequence. Structure. Catalytic site
Model	Task Formulation	Large‐scale multi‐label classification	Level‐wise classification
	Hierarchy Handling	Semantic entailment. Ancestor constraints	Digit‐by‐digit prediction. Tree‐constrained decoding. Hierarchical classifiers
	Zero‐shot Consideration	Required (new GO terms appear)	Rare; EC number relatively stable
Loss	Common Loss Function	Binary Cross‐Entropy Loss. Focal Loss	Binary Cross‐Entropy Loss. Cross‐Entropy Loss
	Additional Constraint	Ancestor consistency loss. Semantic entailment loss	Level‐consistency loss

Beyond predictive accuracy, deep learning models remain large block boxes. While recent efforts, such as DPFunc, have demonstrated the capability to identify potential active sites via attention mechanisms, a critical gap remains in granularity. Existing models often fail to establish a precise correspondence between specific residues and distinct GO terms. Consequently, it remains challenging to distinguish which structural motifs are responsible for which specific GO terms. This lack of explicit residue‐function mapping hinders the translation of model predictions into actionable biological hypotheses, such as guiding directed design of proteins.

Taken together, these challenges highlight the need for more robust and biologically informed computational approaches. Similar issues arise in enzyme function prediction, where hierarchical constraints and annotation sparsity remain central challenges. Next, we will examine computational approaches for EC number prediction and compare them with the GO modeling paradigms discussed above.

## 4 Enzyme Commission Number Prediction

As illustrated in Table 3, EC and GO annotations differ in terms of label characteristics, model architecture, and loss functions. For instance, unlike the loose hierarchical structure of GO terms, EC numbers follow a strictly hierarchical tree structure with four well‐defined levels [14]. In addition, the scale and distribution of the two annotation systems differ substantially. GO contains a significantly larger number of functional labels and exhibits a pronounced long‐tailed distribution, while EC numbers are relatively concentrated, as enzymes directly participate in a limited number of catalytic reactions at a specific region or site [93, 94]. These differences fundamentally shape modeling strategies. GO prediction methods often incorporate multimodal information to compensate for sparse and uneven label distributions, such as sequence, structure, interaction networks, and ontology semantics. In contrast, EC prediction methods tend to focus primarily on protein properties, particularly sequence‐ and structure‐derived catalytic features. Furthermore, in model design, the characteristics of GO necessitate enforcing ancestor–descendant consistency constraints, often through hierarchical regularization or post‐processing strategies. EC‐based methods follow strict tree hierarchy constraints. These differences also influence the design of loss functions. In this section, we first systematically categorize existing methods according to the framework outlined in Section 2, and introduce data processing and modeling strategies in detail for several representative approaches. We further present corresponding application scenarios and summarize the challenges.

### 4.1 Existing Methods

Since enzymes directly catalyze reactions with substrates, existing EC number predictors are more limited compared to GO term prediction frameworks. They primarily rely on protein sequences, structures, ontologies, and LLMs, rather than PPI networks to predict EC functions. Furthermore, annotation data at the substrate‐enzyme or reaction level remains too sparse to support multimodal learning. Accordingly, we review EC prediction methods only within the frameworks currently supported by the literature. The details of all existing approaches can be obtained from Table 4.

**TABLE 4:** The details of existing methods of EC number prediction.

**Methods**	**Encode Schemes & Model Architecture**						**Key Advantages**	**Potential Limitations**
	Sequence	Structure	Ontology	Fusion	LLM	Others		
DeepEC [95]	One‐hot: multi‐1D‐CNN & MaxPooling	/	/	/	/	/	Uses 3 separate CNN modules to classify enzyme presence and predict 3rd/4th level EC numbers.	CNNs can struggle with remote homologs not well represented in training data.
DeepECTransformer [96]	One‐hot: Transformer Encoder (ProtBert) & multi‐1D‐CNN & MaxPooling	/	/	/	/	/	Uses transformer architecture to extract latent features from protein sequences for EC number prediction, which is able to recognize functional motifs.	Some EC number classes show lower accuracy.
CLEAN [97]	One‐hot: pre‐trained ESM‐1b	/	Contrastive Learning	/	/	/	Contrastive learning‐based deep model designed to handle imbalance in EC dataset distributions.	Contrastive framework quality depends on positive/negative sampling strategies.
HIT‐EC [124]	One‐hot: Transformer Encoder	/	/	/	/	/	Hierarchical interpretable transformer model aligning EC prediction architecture with the four‐level EC hierarchy. Introduces training strategy to handle incomplete EC annotations and yields trustworthy evidence‐based predictions.	Computational cost increase as the hierarchical transformer model.
ECPICK [125]	One‐hot: multi‐1D‐CNN & MaxPooling	/	hierarchical layers for four EC digits	/	/	/	Evidential deep learning model that produces biologically interpretable EC predictions with evidence from sequence features (from residue patterns to functions).	CNN and hierarchical classifier architecture is constrained in capturing long‐ranged dependencies compared to transformer models.
GraphEC [99]	One‐hot: pre‐trained ProtTrans	Residue‐distance: Geometric GAT	/	/	/	/	Incorporates label diffusion to solidify predictions via homologous information. Predicts enzyme optimal pH as additional functional context.	Structure prediction quality strongly influences performance.
Clean‐Contact [100]	One‐hot: pre‐trained ESM‐1b	ContactMap: ResNet50	Contrastive Learning	/	/	/	Builds upon CLEAN by integrating protein structural information.	Structure prediction quality strongly influences performance.
TopEC [102]	/	Atom‐KNN: RBF & SBF	/	/	/	/	Incorporates distance and angle features of protein structures and trains a 3D GNN to capture spatial features.	Limited model robustness when binding site uncertainty is high.
MAPred [104]	One‐hot: pre‐trained ESM‐1b & ProtT5	/	Predict different layers step by step with Autoregressive	/	/	/	Multi‐modality, multi‐scale auto‐regressive model combining sequence data and 3D protein tokens to predict EC numbers sequentially, honoring the hierarchical structure.	Auto‐regressive process and dual pathways add complexity and resource consumption.
GloEC [105]	One‐hot: pre‐trained ESM‐1b	/	Graph: Hierarchy GCN	Add & Concatenation	/	/	Captures label correlations bottom‐up and top‐down, improving prediction performance and capturing interactions between EC levels.	Relies on constructed enzyme hierarchy graphs which must be accurate and complete.
EC2Vec [126]	/	/	One‐hot: CNN‐based Autoencoder	/	/	/	Captures label correlations bottom‐up and top‐down, improving prediction performance and capturing interactions between EC levels.	Relies on constructed enzyme hierarchy graphs which must be accurate and complete.
ifDEEPre [127]	One‐hot: pre‐trained LLMs & Self‐guided Attention & CNN Evolutionary Profiles: Self‐guided Attention & CNN	Secondary Structure: Self‐guided Attention & CNN Solvent Accessibility: Self‐guided Attention & CNN	/	/	/	/	Introduces self‐guided attention and short‐cut (residual) connections that improve signal propagation and stabilize training. Offers interpretability by automatically detecting key sequence motifs that contribute to EC prediction.	Predicts only from sequence embeddings and does not integrate 3D structure or active site topology, which can be useful especially for mechanistically driven EC distinctions.
LLaPA [106]	Homology inference: MMseq2 One‐hot: pre‐trained ESM2 as encoder	/	Specical Token for EC number encodings	/	Vicuna‐7b	Molecule: pre‐trained PubChem as encoder	Uses a LLM framework to map protein sequences to EC labels via instruction tuning and multi‐task training, enabling the model to learn deep semantic relationships between sequence patterns and functional categories. Incorporates label semantics and prompt conditioning to help the model understand hierarchical relationships in EC numbering during inference. Designed to be flexible across multiple annotation tasks, enabling joint learning of EC prediction along with other functional properties (e.g., GO terms, binding sites), demonstrating multi‐task capabilities.	As a LLM–based method, it can be susceptible to hallucination or spurious correlations if prompts or task descriptors are ambiguous. The model's performance on strict EC hierarchy accuracy may require careful prompt design and validation, as LLM outputs are not inherently structured.

#### 4.1.1 Sequence‐Based Methods

DeepEC [95] is an early representative deep learning‐based method that utilizes multi‐scale CNNs to extract one‐hot sequence features. It further fuses these features through max pooling operations and finally completes the prediction. Further, its upgraded DeepECTransformer [96] leverages the Bert architecture with enhanced extraction capabilities to extract one‐hot sequence features. Similar to the GO term prediction, pLLMs demonstrate robust predictive capabilities in EC number prediction. CLEAN [97] employs ESM‐1b as the initial sequence feature, utilizes contrastive learning to align representations of enzymes with the same EC numbers, and finally completes classification through unsupervised clustering.

Additionally, evolutionary profiles provide conserved sites and functional domains during the evolutionary process, which are crucial for EC number prediction. HECNet [98] combines PSSM and HMMER evolutionary information with protein sequences and secondary structures to predict EC numbers, achieving promising results.

#### 4.1.2 Structure‐Based Methods

As mentioned before, EC numbers are directly associated with key residues within proteins, so that structural information facilitates the reflection of complex position‐EC relationships. GraphEC [99] first attempts to integrate the active site, optimal temperature, and EC number into a single framework for prediction. It constructs a graph structure based on the distances between $C_{\alpha }$ atoms, utilizing the angles between different amino acids as edge features and the angles between atoms within amino acids as node features. Combined with the amino acid representations from ProtT5 [35], it employs a geometric graph neural network to accomplish various prediction tasks, which enhances accuracy while enabling the prediction of the corresponding active sites.

On the other hand, building upon CLEAN, Clean‐Contact [100] further integrates residue contact maps and utilizes ResNet50 [101] to extract image features, which achieves better performance. In contrast, TopEC [102] attempts to mitigate the impact of structural errors by generating graph features from local coordinate systems. It employs Radial Bessel filters (RBFs) to encode radial distances between atoms and utilizes Spherical Fourier Bessel (SFB) to encode both distances and angles between atoms. Overall, these methods demonstrate that leveraging geometric information within structures to identify key active sites is crucial for EC number prediction.

#### 4.1.3 Ontology‐Based Methods

Since the hierarchy of EC numbers is more strict than that of GO terms, various methods are exploring the use of this ontology constraint to enhance the accuracy and plausibility of prediction results. For example, DeepEC [95] predicts EC numbers step by step. It first predicts whether the target protein is an enzyme, then predicts the first three digit types (class, subclass, sub‐subclass), and finally predicts the complete EC number. Similarly, inspired by autoregressive [103] architecture, MAPred [104] infers the four digits of EC numbers using four prediction heads, with the output from the preceding prediction head serving as input for the subsequent one.

Another strategy involves modeling the hierarchical relationships among EC numbers. GloEC [105] constructs an enzyme taxonomic hierarchy graph based on the inclusion relationships between EC numbers. It then employs a hierarchy‐GCN encoder to fuse protein sequence representations with EC hierarchy graphs, while simultaneously applying L2 regularization loss to ensure that EC numbers with parent‐child relationships exhibit similar representations.

#### 4.1.4 LLM‐Based Methods

Similar to GO term prediction, LLMs can be adapted for enzyme function prediction through fine‐tuning. The general protein LLMs introduced in Section 3.1 can also be applied to EC number prediction using corresponding prompts. However, several studies have found that directly encoding EC logits with “.” as a separator compromises LLM prediction accuracy. To address this, LLaPA [106] introduces a new encoding scheme. It converts “.” token into a symbol more distinguishable in the embedding space, significantly improving the accuracy of EC number prediction with LLMs.

### 4.2 Prediction Scenarios

For EC number prediction, existing common partitioning schemes consist of time‐based splits and similarity‐controlled splits, which is similar with GO term prediction. It should be noted that during data preparation, typically only enzymes with known Level 3 or Level 4 functions are considered. During similarity‐controlled splits, it is important to ensure that as many EC types as possible have training samples to avoid losing valuable training data. Additionally, since many EC numbers lack known enzyme data, few‐shot and zero‐shot prediction are also critical application scenarios in the field of EC number prediction.

### 4.3 Challenges

Currently, EC number prediction still faces numerous challenges. The most significant challenge lies in the extreme imbalance of known sample sizes across functional categories, coupled with the fact that the Level 4 functions of many enzymes remain unknown. This results in poor performance of existing methods for certain EC types.

As the level increases, distinguishing functionally distinct enzymes becomes exponentially difficult. A phenomenon is that minor sequence variations, such as one or two residue mutations, can lead to drastic shifts in catalytic function or substrate specificity. This is particularly evident in large families like carbohydrate‐active enzymes (CAZymes), where high sequence similarity masks diverse EC numbers, challenging the resolution of existing methods.

Furthermore, most existing methods operate in a protein‐centric manner, predicting EC numbers solely based on protein properties while ignoring the substrates. The inability to effectively integrate substrate chemical structures prevents a comprehensive understanding of the enzyme‐substrate interaction landscape, which is essential for precise function assignment.

Finally, similar to GO prediction, the “black‐box” nature of current deep learning models remains a critical limitation. While models may predict an EC number with high confidence, they often fail to identify the specific residues directly involved in the catalytic process (e.g., the catalytic site or binding pocket), which is critical for enzyme redesign [107]. Lacking this mechanistic evidence also makes it difficult for researchers to verify the results experimentally or understand the underlying catalytic machinery.

## 5 Data Source and Evaluation Metrics

### 5.1 Data Source

Above all, existing approaches for functional annotation require multi‐type biological knowledge. In this section, we provide a brief introduction to the relevant databases as follows, where the detailed information can be obtained from Table 5.

**TABLE 5:** Biological databases commonly used in protein functional annotation.

Database	Size	Description	Link
UniprotKB/Swiss‐prot [86]	∼ 574 thousand	Protein sequences and manually reviewed functions	https://www.uniprot.org/uniprotkb?query=reviewed:true
UniprotKB/TrEMBL [86]	∼ 199 million	Protein sequences and automatically predicted functions	https://www.uniprot.org/uniprotkb?query=reviewed:false
Protein Data Bank (PDB) [109]	∼ 246 thousand	Experimentally‐determined 3D structures	https://www.rcsb.org/
AlphaFold DB (AFDB) [110, 111]	∼ 200 million	Predicted 3D structures and MSA profiles	https://alphafold.ebi.ac.uk/
STRING [44]	∼ 28 billion	Known and predicted protein‐protein interactions	https://string‐db.org/
IntAct [112]	∼ 2 million	literature curated or user submitted interactions	https://www.ebi.ac.uk/intact/home
eggNOG [45]	∼ 17 million	Orthology relationships, gene evolutionary histories and functional annotations.	http://eggnog6.embl.de/
Gene Ontology Database [12, 13]	∼ 1 billion	Gene ontology relations and experimentally‐supported annotations	https://geneontology.org/
ENZYME [93]	∼ 7 thousand	Definitions of EC numbers	https://enzyme.expasy.org/
Open Enzyme Database (OED) [128]	∼ 87 thousand	Curated data on enzyme reactions with experimental kinetics parameters	https://openenzymedb.platform.moleculemaker.org/home

UniprotKB [86] is the central hub of functional information on proteins, including protein sequences, descriptions, and their corresponding GO terms and EC numbers. These functions can be categorized into manually reviewed (UniprotKB/Swiss‐prot) [108] and automatically predicted (UniProtKB/TrEMBL) types.

Protein Data Bank (PDB) [109] and AlphaFold DB [110, 111] (AFDB) provide experimentally‐determined structures and predicted structures from AlphaFold2/3, respectively. Notably, current AFDB provides multiple sequence alignment (MSA) of proteins, which are crucial for functional annotation.

STRING [44] and IntAct [112] are two widely used interaction databases covering most proteins, providing multiple interaction types and corresponding confidences. Additionally, eggNOG [45] provides orthology relationships across different species.

The ontologies of GO terms can be obtained from Gene Ontology Database [12, 13], which contains the definitions of each GO term and the relationships between GO terms. And ENZYME Database [93] provides the definitions of enzymes.

### 5.2 Evaluation Metrics

Among tens of thousands of functions, each protein has only a few tens of known functions. Consequently, GO term and EC number prediction can be seen as a multi‐label classification problem. Traditional multi‐label evaluation metrics are used here, including precision (pr), recall (rc), and F1 score, each available in both micro and macro versions:

(1) $$pr_{\mathrm{micro}}=\frac{\sum_{c=1}^{C}{\mathrm{TP}}_{c}}{\sum_{c=1}^{C}\left({\mathrm{TP}}_{c}+{\mathrm{FP}}_{c}\right)}$$

(2) $$rc_{\mathrm{micro}}=\frac{\sum_{c=1}^{C}{\mathrm{TP}}_{c}}{\sum_{c=1}^{C}\left({\mathrm{TP}}_{c}+{\mathrm{FN}}_{c}\right)}$$

(3) $$F1_{\mathrm{micro}}=\frac{2\cdot pr_{\mathrm{micro}}\cdot rc_{\mathrm{micro}}}{pr_{\mathrm{micro}}+rc_{\mathrm{micro}}}$$

(4) $$pr_{\mathrm{macro}}=\frac{1}{C}\sum_{c=1}^{C}\frac{{\mathrm{TP}}_{c}}{{\mathrm{TP}}_{c}+{\mathrm{FP}}_{c}}$$

(5) $$rc_{\mathrm{macro}}=\frac{1}{C}\sum_{c=1}^{C}\frac{{\mathrm{TP}}_{c}}{{\mathrm{TP}}_{c}+{\mathrm{FN}}_{c}}$$

(6) $$F1_{\mathrm{macro}}=\frac{1}{C}\sum_{c=1}^{C}\frac{2\cdot {\mathrm{TP}}_{c}}{2\cdot {\mathrm{TP}}_{c}+{\mathrm{FP}}_{c}+{\mathrm{FN}}_{c}}$$

where C denotes the total number of function classes, $TP_{c}$, $FP_{c}$, and $FN_{c}$ are the corresponding true positives, false positives and false negatives, respectively. Micro‐averaged metrics reflect the overall prediction performance and are dominated by frequency of functions, while macro‐averaged metrics assign equal importance to all functions and are therefore more sensitive to the prediction performance on rare and long‐tail distributions.

Further, to evaluate the results more comprehensively, some explored metrics have been proposed, including Fmax and AUPR under different weighting schemes, where each weighting scheme reflects distinct functional attributes [2]. Fmax is the maximum of F1 score across all possible thresholds, determined jointly by the corresponding precision (pr) and recall (rc):

(7) $$pr\left(t\right)=\frac{1}{\left\|n(t)\right\|}\sum_{i\in n(t)}\frac{\sum_{j\in C}I(P_{i}\left(j\right)\geq t\wedge j\in T_{i})}{\sum_{j\in C}I(P_{i}\left(j\right)\geq t)}$$

(8) $$rc\left(t\right)=\frac{1}{\left\|n\right\|}\sum_{i\in n}\frac{\sum_{j\in C}I(P_{i}\left(j\right)\geq t\wedge j\in T_{i})}{\sum_{j\in C}j\in T_{i}}$$

(9) $$F1=\frac{2\times pr(t)\times rc(t)}{pr(t)+rc(t)}$$

where t is the selected threshold, n is the total number of proteins, and $\left\|n(t)\right\|$ is the set of proteins with at least one predicted function at the corresponding threshold. $P_{i}\left(j\right)$ is the predicted probability of protein i for function j. $T_{i}$ is the true function set of protein i. In summary, Fmax is the maximum F1 score when t ranges from 0.01 to 0.99, while AUPR denotes the area under the precision curve relative to the x‐axis at different recall values.

Additionally, each function (GO term/EC number) corresponds to a different number of known samples. “Child” functions represent more specific content compared to their ancestors. It is more meaningful and challenging to accurately predict rare and detailed functions. Consequently, based on the above metrics, different weighting schemes are introduced to highlight the differences among various functions:

(10) $$IC_{j}=\log_{2}\frac{1}{prob(j|parent(j))}$$

(11) $$DP_{j}=IC_{j}\times \log_{2}\left(depth(j)+1\right)$$

where prob(j|parent(j)) denotes the probability that function j co‐occurs with its parent node, depth(j) is the shortest distance from j to the root node. IC describes the information content of each function, with higher being more valuable [2]. DP further incorporates the depths of functions, where deeper depth indicates more specific functions [4]. These weighting schemes are widely used for GO term evaluation and can be easily integrated with common precision and recall, thereby constructing corresponding weighted Fmax and AUPR scores:

(12) $$wpr\left(t\right)=\frac{1}{\left\|n(t)\right\|}\sum_{i\in n(t)}\frac{\sum_{j\in C}w\left(j\right)\times I(P_{i}\left(j\right)\geq t\wedge j\in T_{i})}{\sum_{j\in C}w\left(j\right)\times I(P_{i}\left(j\right)\geq t)}$$

(13) $$wrc\left(t\right)=\frac{1}{\left\|n\right\|}\sum_{i\in n}\frac{\sum_{j\in C}w\left(j\right)\times I(P_{i}\left(j\right)\geq t\wedge j\in T_{i})}{\sum_{j\in C}w\left(j\right)\times j\in T_{i}}$$

(14) $$w\left(j\right)\in \left\{IC_{j},DP_{j}\right\}$$

Based on the information content of functions, another metric, $S_{min}$ (the minimum of S‐score), is utilized for measuring semantic distance between the real and predicted functions:

(15) $$ru\left(t\right)=\frac{1}{\left\|n\right\|}\sum_{i\in n}\sum_{j\in C}IC_{j}\times I\left(P_{i}\left(j\right)\leq t\wedge j\in T_{i}\right)$$

(16) $$mi\left(t\right)=\frac{1}{\left\|n\right\|}\sum_{i\in n}\sum_{j\in C}IC_{j}\times I\left(P_{i}\left(j\right)\geq t\wedge j\notin T_{i}\right)$$

(17) $$S_{min}=\underset{t}{min}\sqrt{ru{\left(t\right)}^{2}+mi{\left(t\right)}^{2}}$$

where ru denotes the remaining uncertainty and mi is misinformation.

Notably, as known EC numbers are not always complete, it is necessary to evaluate each level independently. For example, when evaluating the third level, the digits at the fourth level will be ignored. At this point, EC 1.2.2.3 and EC 1.2.2.5 are considered as the same class, denoted as EC 1.2.2.x.

## 6 Future Directions and Conclusions

**FIGURE 4:**
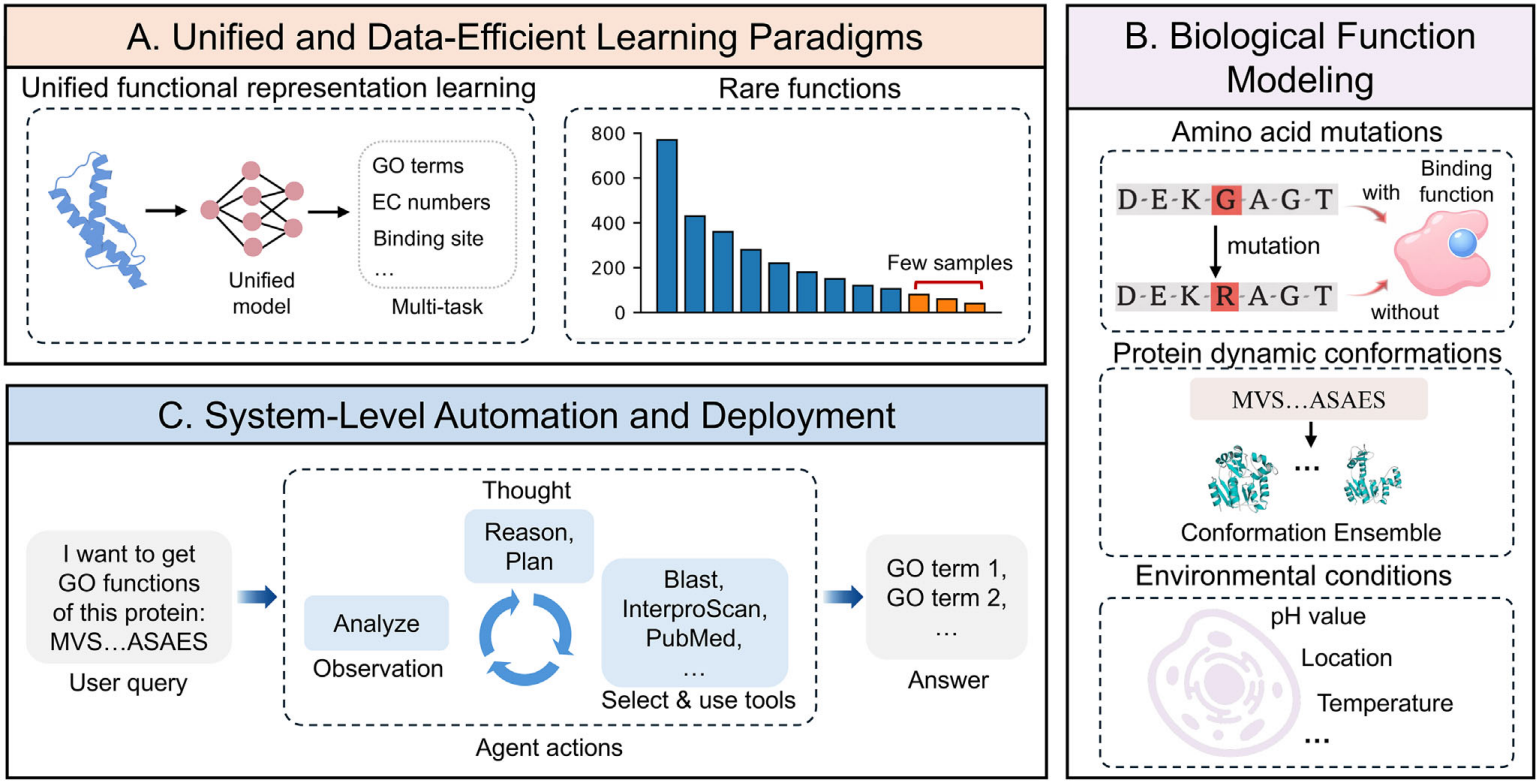
Future directions, including: (A) Unified and Data‐Efficient Learning Paradigms. (B) Biologically Function Modeling. (C) System‐Level Automation and Deployment.

Despite remarkable advances in AI‐driven protein functional annotation, fundamental challenges remain that limit the generalizability, biological realism, and scalability of current approaches. Specifically, we illustrate these challenges from three aspects (Figure 4): (I) unified and data‐efficient learning paradigms; (II) biologically function modeling; (III) system‐level automation and deployment. These dimensions reflect a progressive transition from improving foundational learning architectures to redefining protein function in dynamic biological contexts, and ultimately to integrating these capabilities into autonomous annotation systems.

### 6.1 Unified and Data‐Efficient Learning Paradigms

At the methodological level, improving generalization across heterogeneous annotation systems and long‐tailed function distributions remains a primary challenge, which consist of two critical frontiers: (I) proposing a unified representation learning framework for multi‐task functional annotation; (II) addressing the data scarcity inherent in rare function annotation.

Unified functional representation learning aims to jointly embed GO terms and EC numbers within a shared latent space, enabling models to capture the complementary biological semantics of the two annotation systems. Moreover, protein function is not only reflected at the ontology level but also at the structural and mechanistic levels. Ligand‐binding sites, such as catalytic residues and substrate‐recognition pockets, are intrinsically linked to MF terms and EC annotations, as enzymatic activity fundamentally depends on the ability to bind and transform specific ligands [113]. However, current function predictors [10, 72, 114, 115] typically treat them as separate tasks and are developed in isolation, even though these two ontologies represent similar or related functions (e.g., “GO:0016491”‐ “oxidoreductase activity” and “EC:1.x.x.x”‐ “oxidoreductase”). Although some works like DeepFRI employ the same architecture to predict GO and EC, they remain independently trained models, which prevents the transfer of functional clues across different annotation levels. A unified multi‐task framework would allow models to share information across these related objectives, exploit cross‐level semantic dependencies, and improve predictions for fine‐grained and rarely observed functions. Future research may utilize a unified representation framework to align cross‐ontologies to shared embedding spaces, aiming to support multi‐task functional annotation and provide a more comprehensive understanding of protein functions.

Rare functions are typically annotated on only a few proteins. Accurately predicting these rare functions is both highly significant and challenging. Current models, particularly those trained in classification paradigms, tend to overfit common functions while failing to generalize to rare or newly functions. Only a few approaches have utilized zero‐shot or few‐shot strategies to mitigate this limitation, but their performance remains insufficient. In future, incorporating more predefined biological knowledge and employing more efficient multimodal fusion techniques may mitigate this limitation. For instance, integrating functional text content into the learning and inferring processes, or leveraging the powerful knowledge learning capabilities of LLMs through prompt fine‐tuning for functional annotation tasks.

### 6.2 Biologically Function Modeling

Beyond architectural improvements, a deeper conceptual shift is needed to model protein function as a dynamic and context‐dependent phenomenon, which is critical for high‐resolution functional characterization. Accordingly, we summarize these challenges from three complementary perspectives: molecular determinants, conformational dynamics, and environmental context: (I) deciphering the functional variations of amino acid mutations; (II) integrating protein conformational dynamics into predictive models; (III) developing frameworks for functional annotation with environmental conditions.

Amino acid mutations easily affect both protein structure and function. This is a common strategy used in enzyme engineering to optimize enzymes' functional activity. However, according to our research, existing methods are not sensitive to single or multiple amino acid mutation perturbations. AnnoPro is the sole method that shows superior sensitivity to mutational effects, accurately capturing mutation‐induced loss of functions in two case studies. Future research should develop more efficient models capable of integrating mutation‐aware mechanistic modeling, including structural perturbation simulations, catalytic pocket reconfiguration, and energy landscape shifts.

Protein dynamic conformations are ubiquitous and closely related to functions. But existing experimental or predicted structures are static. Additionally, due to the high computational cost of molecular dynamics (MD) simulations, current methods predict functions based on a single structural state, preventing the prediction of functions across diverse structural conformations. Currently, several methods have been proposed for dynamic conformation generation using deep learning [116, 117, 118]. These methods are trained on data generated by MD simulations or dynamic NMR structures; thus, they can rapidly infer structural conformations in different states, holding promise for future applications in protein functional annotation.

Environmental conditions are necessary for proteins, especially enzymes, to perform their functions, such as cell type, subcellular location, and the pH, temperature, and substrate required by the enzymes. Existing functional annotation methods primarily rely on static and isolated biological contexts. Integrating conditional parameters in future work will facilitate the high‐precision elucidation of protein functions and enzymatic catalytic processes.

### 6.3 System‐Level Automation and Deployment

As predictive capabilities mature, the next frontier lies in transforming isolated models into integrated, autonomous annotation systems. It is necessary to design domain‐specific agents for automated protein functional annotation.

Agents represent an emerging paradigm that extends large language models and existing tools into automated problem‐solving frameworks capable of completing complex tasks through multi‐step reasoning and tool usage. Typically, the operations of agents can be divided into four stages: task and plan specification, tool selection, tool execution, and result analysis. Benefiting from the strong comprehension, fast reasoning, and summarization abilities of LLMs, such agents can act as domain experts, often achieving human‐expert‐level or even superior performance in specific application scenarios.

However, domain‐specific agent design requires professional workflows that are tightly coupled with domain‐specific resources and computational tools. Recently, while some agents have been proposed in various areas (e.g., ChemCrow [119] in chemistry, SpatialAgent [120] in spatial biology), agent frameworks tailored for protein functional annotation remain unexplored. Designing agents that can integrate LLMs (e.g., GPT [54], DeepSeek [55]) with protein‐specific tools (e.g., D‐I‐TASSER [121] and DPFunc [10]) and databases (e.g., InterPro [26] and Pfam [27]) presents significant methodological and engineering challenges. It is quite necessary to develop protein‐centric agents for functional annotation, enabling them to automatically perform sequence analysis, structural modeling, ontology reasoning, and evidence integration to generate reliable and interpretable functional annotation. The development of protein‐centric agents can accelerate large‐scale annotations, experimental design, and principal exploration, opening a new path for fully automated, expert‐level protein functional annotation.

Collectively, AI‐based protein function annotation has progressed rapidly, driven by advances in deep learning architectures, massive biological datasets, and ontology‐guided frameworks. Looking ahead, future frameworks are encouraged to integrate more multimodal biological knowledge, more powerful mechanistic modeling, and the reasoning capabilities of LLMs to build a unified representation model that further improves the accuracy of rare function prediction and mutation‐induced functional shift detection. Concurrently, achieving high‐resolution functional annotation under dynamic conformational states and varying environmental conditions represents a pivotal research frontier. Ultimately, accurate and comprehensive functional annotation constitutes the bedrock for reliable protein redesign and the acceleration of drug development.

## Author Contributions

M.L. supervised the research. W.W. and M.L. conceptualized the study. W.W. wrote the original draft. W.W. and Q.Y. drew figures. W.W., Q.Y., M.Z., R.Z., and M.L. participated in discussions. All authors wrote the manuscript and approved the final version.

## Conflicts of Interest

The authors declare no conflicts of interest.

## Data Availability

The authors have nothing to report
